# The Effect of EECP on Ischemic Heart Failure: a Systematic Review

**DOI:** 10.1007/s11886-023-01943-1

**Published:** 2023-08-29

**Authors:** Ling Xu, Ming Cui, Wei Zhao

**Affiliations:** 1 Department of Cardiology and Institute of Vascular Medicine, Peking University Third Hospital, Beijing, 100191 China; 2 State Key Laboratory of Vascular Homeostasis and Remodeling, Peking University, Beijing, 100191 China; 3 NHC Key Laboratory of Cardiovascular Molecular Biology and Regulatory Peptides, Peking University, Beijing, 100191 China; 4 Beijing Key Laboratory of Cardiovascular Receptors Research, Beijing, 100191 China

**Keywords:** Enhanced external counterpulsation, Ischemic heart disease, Cardiac function, Mechanism

## Abstract

**Purpose of Review:**

Heart failure is a serious global health problem, and coronary artery disease is one of the main causes. At present, the treatment options for ischemic heart failure (IHF) are limited. This article mainly aims to explore the evidence of enhanced external counterpulsation (EECP) as a non-invasive cardiac rehabilitation method in patients with IHF and to make a preliminary exploration of its mechanisms.

**Recent Findings:**

According to the existing evidence, the standard course of EECP is safe in patients with IHF and can significantly improve the quality of life of these patients. The effect of EECP on systolic function is still unclear, while EECP has a significant improvement effect on cardiac diastolic function. At the same time, this treatment can reduce the re-hospitalization rate and emergency visit rate of patients within 6 months. In terms of mechanisms, in addition to the immediate hemodynamic effect, existing evidence mostly suggests that its improvement of cardiac function may come from its upregulation of shear stress to improve myocardial perfusion.

**Summary:**

EECP is safe to use in patients with stable ischemic heart failure, and it can improve the performance status of patients and may be beneficial to cardiac function and reduce the short-term re-hospitalization rate.

## Introduction

With the progress of population aging, heart failure has gradually become an important health problem in today’s world, causing huge social and economic burden. In recent years, although the incidence of heart failure has decreased compared with before under active prevention and control, the mortality rate is still on the rise [1]. The 2020 China Heart Failure Medical Quality Control Report pointed out that coronary heart disease (CHD) is one of the main causes of heart failure in China, accounting for 48.3% [2]. Therefore, the diagnosis and treatment of ischemic heart failure (IHF) is urgent. The enhanced external counterpulsation (EECP) is a safe and effective cardiac rehabilitation treatment. Its unique dual-pulsed blood flow effect can increase immediate coronary perfusion, reduce cardiac afterload, and relieve myocardial ischemia [3]. It has now been recommended for use in patients with refractory angina [4, 5]. Regarding the role of EECP in patients with IHF, previous researchers have focused more on the improvement of ischemic symptoms in such patients. It was found that EECP can also significantly improve ischemic symptoms in IHF patients [6–8]. Therefore, in 2002, the US FDA approved the application of EECP to patients with angina pectoris combined with heart failure. This article intends to summarize and analyze the literature on the impact of EECP on the cardiac function of patients with IHF and its related mechanisms.

## Efficacy

There are only a few randomized controlled studies on the clinical efficacy of EECP in patients with IHF, most of which are cohort studies, and the sample sizes included are relatively small (see Table 1 for details).

**Table 1:** Characteristics of research included only ischemic heart failure patients

Study	Type of study	Period of follow-up	Sample size	Male (%)	Age, mean (SD or range)	LVEF, mean (SD or range)	Main indicators	Main outcomes
Ozlem et al. 2007 [9]	Prospective cohort study	Before and 6 months after the EECP therapy	450	81	69 ± 11	30 ± 8%	Emergency room visits Hospitalizations	The mean number of ED visits per patient and hospitalizations was significantly reduced
Yavari et al. 2007 [15]	Retrospective cohort study	Before and after the termination of the EECP protocol	67	70.1	63.70 ± 9.62	46.48 ± 13.49	LVEF LVESD LVEDD Exercise test duration CCS functional class	LVEF, LVESD, and LVEDD were not significantly changed after the therapy, while exercise test duration and CCS functional class were significantly improved
Maryam et al. 2009 [16]	Prospective cohort study	Before and after the termination of the EECP protocol	20	65	63 ± 9	40.25 ± 12.72%	LVEF GLS E/A E/Em	The above parameters were all significantly improved after the EECP therapy
Fariba et al. 2012 [17]	Prospective cohort study	Before and 1 year after the EECP therapy	50	68	NM	43.55 ± 11.60	LVEF LVEDD LVESD Exercise duration Ischemia severity grading Extent of ischemic myocardium Extent of infracted myocardium	Except for the extent of ischemic myocardium, other parameters were significantly improved 1 month after the therapy. However, there was no further improvement in the above parameters 1 year after the therapy
Darren et al. 2015 [24]	RCT	Before and after the termination of the EECP protocol	17 (E: *N* = 10; C: *N* = 7)	E (80), C (71)	E (64.2 ± 2.6), C (66.2 ± 3.5)	E (35.1 ± 4.6), C (34.3 ± 4.2)	AIx AoPW LVEw Myocardial oxygen demand	The above parameters were all significantly improved after the EECP therapy
Ramasamy et al. 2016 [18]	Retrospective cohort study	Before and after the termination of the EECP protocol	72	87.5	58	43.97 ± 15.97	AIx LVEF	The LVEF was significantly improved after the therapy. Aix was only significantly improved after the therapy in the patient which had baseline brachial systolic pressure > 100 mmHg
Kristen et al. 2016 [11]	Prospective cohort study	Before and after the termination of the EECP protocol	99	72	68.2 (60.5, 73.9)	NM	90-day readmission rates 6-min walk tests NYHA	The above parameters were all significantly improved after the EECP therapy
Amr et al. 2018 [13]	Prospective cohort study	Before and 3 months after the EECP therapy	42 (E: *N* = 20; C: *N* = 22)	E (40), C (36.4)	E (55.25 ± 7.69), C (59.64 ± 8.2)	E (40.85 ± 5.29), C (43.00 ± 6.05)	LVEF CCS functional class NYHA	Except for the LVEF, other parameters were significantly improved 3 months after the therapy

*RCT* randomized controlled trial, *EECP* enhanced external counterpulsation, *E* EECP group, *C* control group, *NM* not mentioned, *LVEF* left ventricular ejection fraction, *GLS* global longitudinal strain, *LVEDD* left ventricular end-diastolic dimension, *LVESD* left ventricular end-systolic dimension, *AoPW* central aortic pressure waveform, *LVEw* left ventricular wasted energy, *NYHA* New York Heart Association, *CCS* Canadian cardiovascular society, *ED* emergency department

### EECP Effects on the Performance Status of IHF Patients

In 2006, Arthur et al. published the results of the PEECH study, which included 130 IHF patients with NYHA class II–III, who were randomly assigned to the EECP group or the control group. It was found that the NYHA classification and quality of life of the EECP group were significantly improved after treatment. In terms of exercise tolerance, the total exercise time of the EECP group was significantly higher than that of the control, and this effect lasted for 6 months after treatment. Improvement in peak oxygen uptake (VO_2_peak) was also observed in the EECP group 1 week after treatment, but this effect disappeared during follow-up [9]. Subsequent researchers conducted subgroup analysis based on age and defined an increase in exercise time by 60 s and an increase in VO_2_peak by 1.25 ml/kg/min as exercise response. It was found that in subjects over 65 years old, both exercise response rates were significantly higher in the EECP group. The above differences may be due to weaker baseline exercise capacity in elderly patients, which may make it easier to demonstrate the benefits of intervention [10]. Since then, some small sample cohort studies have been conducted one after another, and the results are consistent with the PEECH study. In a word, after the standard course of EECP treatment, total exercise time, 6-min walk test, and NYHA classification were significantly improved in patients with IHF, and these effects did not vary with the difference in baseline ejection fraction levels [8, 11–14].

### EECP Effects on Systolic Function of IHF Patients

Regarding changes in objective indicators of systolic function, left ventricular ejection fraction (LVEF) is the most common evaluation index. However, there is no unified conclusion on the effect of EECP on LVEF. Amr et al. included 42 patients with IHF after coronary artery bypass surgery, and no improvement in LVEF was found after 3 months of follow-up after the treatment [13]. Yavari et al. also did not find a significant improvement in LVEF after EECP treatment in 67 patients with IHF (46.48 ± 13.49% vs. 48.14 ± 16.03, NS) [15]. However, a small sample cohort study conducted by Maryam et al. (*N* = 20) showed that LVEF improved significantly after standard EECP treatment (40.25 ± 12.72% vs. 46.25 ± 12.97%, *P* < 0.01) [16]. Fariba followed up 50 patients with IHF who underwent EECP treatment for 1 year and found a significant improvement in LVEF, but in subgroup analysis the efficacy was only observed in patients with baseline LVEF < 40% [17]. While in Ramasamy’s study, the above conclusions did not appear; they included 72 CHD patients with left ventricular dysfunction and found that regardless of the baseline LVEF; this parameter was all improved after EECP treatment [18].

The reasons for the different results above might be affected by the heterogeneity of the included patients, including the differences in the degree of coronary artery lesion, revascularization strategy, and baseline LVEF, which may affect the efficacy of EECP. In addition, for patients with preserved or mildly decreased ejection fraction, whether LVEF is an appropriate evaluation index is also worth exploring. In recent years, the development of cardiac speckle-tracking technology has been rapid. Its myocardial strain parameters can more sensitively and comprehensively evaluate myocardial systolic function than LVEF. A small amount of research has applied speckle-tracking technology to evaluate the clinical efficacy of EECP in CHD patients without heart failure and found significant improvement in global longitude strain (GLS) during EECP treatment [16, 19]. Therefore, it may be necessary to comprehensively evaluate the effect of EECP on cardiac systolic function in patients with IHF by more sensitive indicators on the basis of standardizing the subjects.

### EECP Effects on Diastolic Function of IHF Patients

As for the impact of EECP on cardiac diastolic function, there are fewer relevant studies, but the conclusions are relatively consistent [14, 16, 20]. Maryam et al. included 20 angina patients in NYHA functional class III–IV and evaluated diastolic function by E/A (0.92 ± 0.41 vs. 1.08 ± 0.46, *P* < 0.05) and E/Ea (12.61 ± 4.22 vs. 15.44 ± 6.96, *P* < 0.05); the above indicators improved significantly after EECP treatment. Other researchers evaluated patients with CHD without heart failure using E/A, left ventricular end-diastolic pressure, peak filling rate (PFR), and the time to PFR, and found that EECP has a potential positive effect on cardiac diastolic function.

### EECP Effects on Prognosis of IHF Patients

In the PEECH study, the effect of EECP on short-term prognosis was evaluated; 9% of patients underwent EECP and 13.6% of controls were readmitted within 180 days [9]. Kristen M. et al. enrolled 99 IHF patients who were readmitted for heart failure and gave them EECP treatment after discharge to evaluate the readmission rate within 90 days; only 6 patients (6.1%) had unplanned readmissions compared to the predicted 34%. The above results suggested that EECP might reduce the short-term all-cause readmission rate of IHF patients [11]. In a study of 450 patients with refractory angina and left ventricular dysfunction (LVEF < 30 ± 8%), EECP significantly reduced 6-month emergency room visits by 78% and hospitalizations by 73% [7]. Although the above studies seem to suggest that EECP has some improvement effect on the prognosis of IHF, the heterogeneity of the existing studies is obvious, the observation indicators are relatively single, and they all limit the credibility of the results. In the future, it is necessary to further expand the sample size and extend the follow-up time.

## Safety

Given the hemodynamic changes during EECP treatment, an increase in the amount of blood returning to the heart might worsen congestive heart failure. Hence, the evaluation of the safety of EECP in IHF patients is extremely important. However, in clinical practice, no increase in heart failure exacerbation or thrombotic events was observed during or after EECP treatment. The safety of elderly subjects was consistent with that of the entire study population. However, discomfort such as skin and bone occurred significantly more frequently than in the control group [9, 10].

## The Potential Mechanisms by Which EECP Improves IHF

**Fig. 1:**
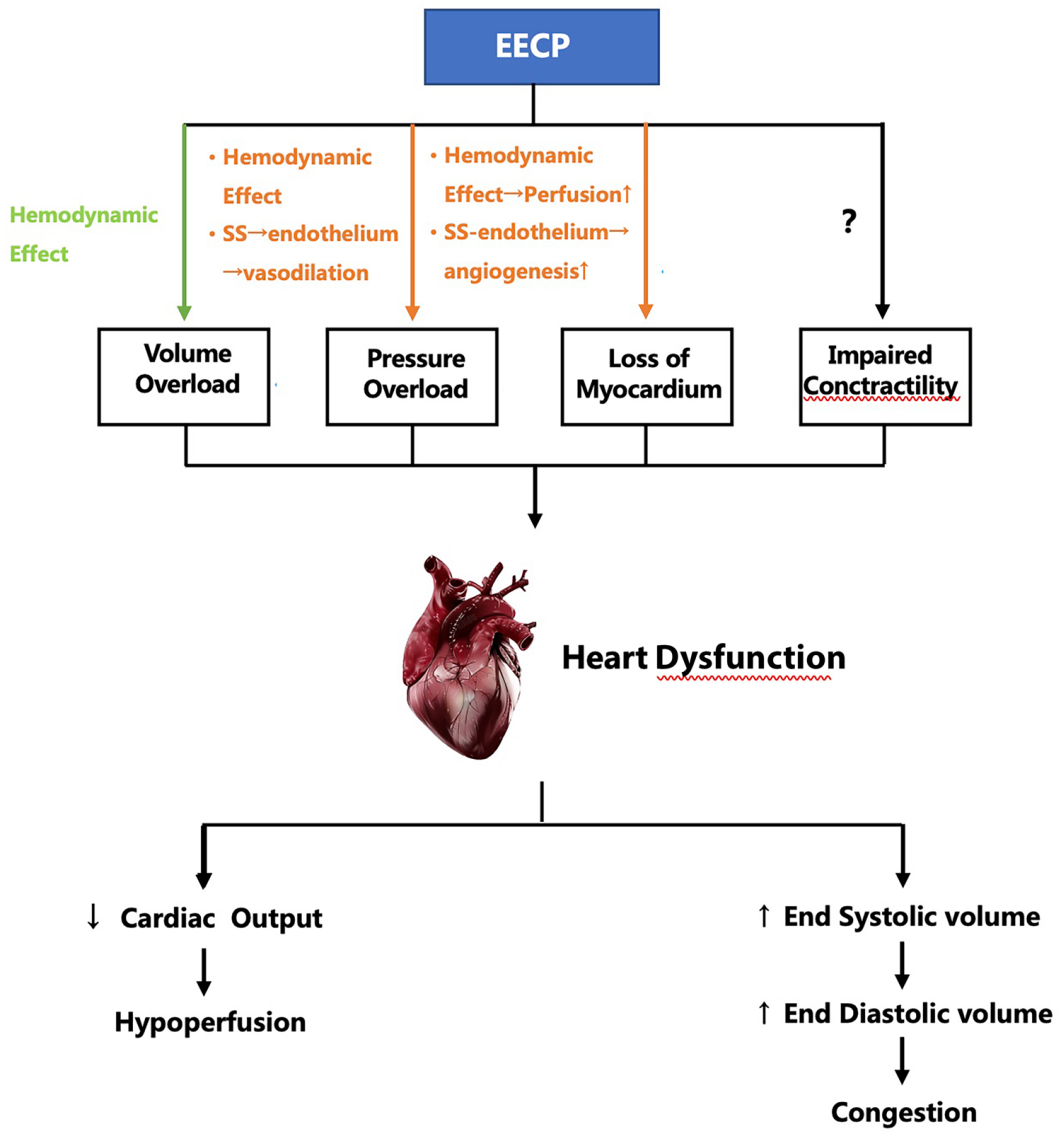
The potential mechanisms by which EECP improves heart failure. EECP, enhanced external counterpulsation; SS, shear stress; green arrow: may be harmful; orange arrow: helpful

At this stage, the mechanisms of EECP are mainly divided into immediate hemodynamic effects and long-term anti-ischemic mechanisms mediated by shear stress, and there are still other potential mechanisms that need to be explored (see Fig. 1 for details).

### Reducing Cardiac Load

EECP has significant hemodynamic effects. The synchronous release of all cuffs during systole can reduce systolic blood pressure by 9–16 mmHg, thereby reducing cardiac afterload [6]. In addition, lots of research has shown that EECP can improve shear stress, which can act on endothelial cells, increasing the expression of vasodilators such as NO and decreasing vasoconstrictors such as ET-1 [21, 22, 23•], so the endothelial cell-dependent vasodilation effect increases, further reducing peripheral blood pressure. Darren and other researchers used pulse wave analysis technology and found that the left ventricular energy consumption index and myocardial oxygen demand decreased after EECP treatment [24]. Other researchers observed a significant decrease in resting heart rate after EECP treatment [7, 14]. The above results further indicate a reduction in cardiac load after EECP treatment.

### Reducing the Loss of Myocardium

For IHF, the loss of myocardium is mainly due to ischemia. Combined with the existing literature, EECP improves ischemia mainly through the following two mechanisms. One is through direct hemodynamic effects; sequential inflation of EECP cuffs during diastole increases the blood flow at the root of the aorta, raising diastolic blood pressure levels by 26—157% and improving coronary perfusion [6]. And the other mechanism is through improving shear stress to exert subsequent effects. The damage of endothelial cells is the initiation of atherosclerotic lesions, and endothelial cells are also the main responsive cells to shear stress. They can sense and transduct the mechanical signals, initiating mechanical biological signal conversion and feedback mechanisms, ultimately changing endothelial cell morphology and function [25]. Firstly, shear stress can promote the growth of new endothelial cells by directly acting or promoting directional differentiation of endothelial progenitor cells and inhibiting their excessive proliferation and apoptosis [26–28]. Secondly, as mentioned above, shear stress can affect the secretion of vasoactive substances by endothelial cells, thereby promoting coronary artery dilation [25, 29, 30]. Thirdly, shear stress can regulate inflammatory factors and oxidative stress and slow down the progression of atherosclerosis and stabilize plaques, reducing the occurrence of acute coronary events. Finally, vascular endothelial growth factor and angiopoietin which involved in regulating vascular generation can respond to changes in shear stress [31–35]. The proliferation, differentiation, and tube formation of endothelial progenitor cells are also regulated by shear stress [36–38]. Therefore, shear stress can relieve the ischemia by promoting angiogenesis. Buschmann et al. selected 23 patients with stable angina pectoris and divided them into EECP group and control group; coronary angiography was performed before and after treatment. It was found that the collateral flow index (CFI) and fractional flow reserve (FFR) in the EECP group were significantly improved. The above studies indicate that EECP treatment can clearly improve myocardial perfusion, and its mechanism may be related to increasing collateral circulation [39].

### Enhancing the Contractility

As mentioned earlier, EECP can enhance cardiac contractility by improving myocardial ischemia. However, there is no study to evaluate whether EECP can directly affect the myocardial tissue. Mitochondria are important places for cells to perform respiration and oxidative phosphorylation, and finally produce ATP. Cardiac mitochondria provide continuous energy for the beating heart, and its functional state directly affects cardiac contractility. Recent works of authors’ research group suggested that standard course of EECP treatment may increase plasma adrenomedullin (ADM) level. ADM is a vasodilator peptide which secreted by endothelium. Previous animal studies have found that the absence of ADM action could induce myocardial fibrosis and decrease LVEF, accompanied by a decrease in mitochondrial number, membrane potential, and respiratory function [40•]. At the same time, previous studies have suggested that ADM can affect the L-type calcium current in guinea-pig ventricular myocytes, and calcium ion level is an important factor affecting myocardial contractility, which is the basis for myocardial electromechanical coupling [41]. Therefore, we are curious whether EECP can directly increase cardiac contractility by improving mitochondrial function or affecting electromechanical coupling through upregulating ADM or other substances, and whether this process is mediated by shear stress. However, the existing evidence is not enough to support the above hypothesis. Further studies are needed to clarify in the future.

## Summary

Given the mechanisms of EECP, the primary concern for its application in patients with heart failure is safety assessment. Based on existing research results, the use of EECP in the stable stage of ischemic heart failure does not increase the risk of serious adverse events (including worsening heart failure and thrombotic events), but the discomforts of skin and bone may increase. In terms of effectiveness, EECP treatment can significantly improve NYHA classification and increase exercise time regardless of the cause of heart failure or baseline LVEF. At the same time, there is a clear improvement in diastolic function of the heart, while the effect on systolic function is not clear. There is an improvement in short-term prognosis indicators such as heart failure readmission after EECP treatment. In terms of exploring the mechanisms by which EECP improves heart failure, in addition to its immediate hemodynamic effects to reduce cardiac afterload and increase coronary perfusion, existing evidence mainly suggests that EECP can improve endothelial cell function to increase vasodilator release and promote neovascularization by upregulating shear stress and thus increasing myocardial contractility.

Combining the above conclusions, it can be found that there are still many limitations in the exploration of EECP application in IHF: ① lack of large-scale RCT studies, existing research designs have shortcomings such as small sample size, non-blinding, high heterogeneity, which affect the reliability of results; ② lack of long-term follow-up observation limits the long-term evaluation of safety and efficacy of EECP in patients with IHF; ③ the evaluation indicators are relatively single, the comprehensive application of echocardiography, speckle-tracking imaging, cardiac magnetic resonance, and other methods can more comprehensively and sensitively evaluate changes in cardiac function; ④ in terms of exploring the mechanisms, it is currently mainly limited to its improvement in myocardial perfusion. Future research would explore whether EECP can directly enhance myocardial cell contractility to improve cardiac function.

## Data Availability

Data availability is not applicable to this article as no new data were created or analyzed in this study.
